# ß-Adrenoreceptors in Human Cancers

**DOI:** 10.3390/ijms24043671

**Published:** 2023-02-12

**Authors:** Zoltan Kraboth, Bernadette Kalman

**Affiliations:** 1 Department of Pathology, School of Medicine, University of Pécs, 12. Sziget Street, 7624 Pécs, Hungary; 2 Szentagothai Research Center, University of Pécs, 20. Ifjusag Street, 7624 Pécs, Hungary; 3 Department of Laboratory Medicine, School of Medicine, University of Pécs, 13. Ifjusag Street, 7624 Pécs, Hungary

**Keywords:** ß-adrenoreceptors, adrenergic signaling, ß-blockers, cancer, stress, physiological pathways hijacked by cancer

## Abstract

Cancer is the leading cause of death and represents a significant economic burden worldwide. The numbers are constantly growing as a result of increasing life expectancy, toxic environmental factors, and adoption of Western lifestyle. Among lifestyle factors, stress and the related signaling pathways have recently been implicated in the development of tumors. Here we present some epidemiological and preclinical data concerning stress-related activation of the ß-adrenoreceptors (ß-ARs), which contributes to the formation, sequential transformation, and migration of different tumor cell types. We focused our survey on research results for breast and lung cancer, melanoma, and gliomas published in the past five years. Based on the converging evidence, we present a conceptual framework of how cancer cells hijack a physiological mechanism involving ß-ARs toward a positive modulation of their own survival. In addition, we also highlight the potential contribution of ß-AR activation to tumorigenesis and metastasis formation. Finally, we outline the antitumor effects of targeting the ß-adrenergic signaling pathways, methods for which primarily include repurposed ß-blocker drugs. However, we also call attention to the emerging (though as yet largely explorative) method of chemogenetics, which has a great potential in suppressing tumor growth either by selectively modulating neuronal cell groups involved in stress responses affecting cancer cells or by directly manipulating specific (e.g., the ß-AR) receptors on a tumor and its microenvironment.

## 1. Introduction

Cancers are among the leading causes of death and bestow a huge economic burden worldwide. The numbers of cases continuously rise as a result of population growth, increased life expectancy, and the adoption of certain lifestyle choices in the most developed countries [1].

**Figure 1:**
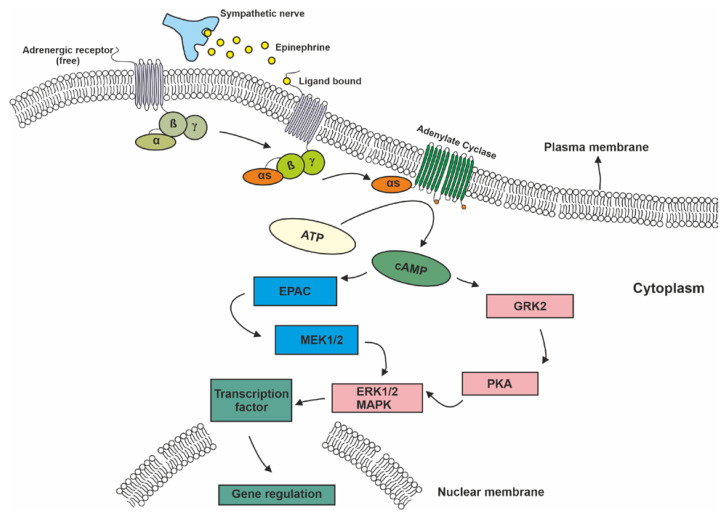
Main pathways of ß-adrenergic signaling. The figure above schematically depicts what pathways are involved in carrying signals to the nucleus upon engagement of catecholamine ligands with their adrenergic receptors. (Abbreviations: cAMP, cyclic AMP; PKA, proteinase K; GRK2, adrenergic beta receptor kinase; EPAC, guanine nucleotide exchange protein; ERK1/2, extracellular signal-regulated kinase; MEK1/2, mitogen activated protein kinase kinase 1/2).

In addition to the well-known risk factors (smoking, overweight, physical inactivity, etc.), psychosocial factors such as stress, chronic depression, and a lack of social support also contribute to cancer development and progression [2]. One way to cope with stress is through the activation of the sympathetic nervous system (SNS) and the release of cortisol and catecholamine neurotransmitters such as adrenaline and noradrenaline (alternatively called epinephrine (E)] and norepinephrine (NE), respectively). The effects of these monoamines are mediated through interactions with the α- and ß-ARs [3]. Nine different ARs are encoded in the human genome, and they can be separated into three distinct subfamilies of α1, α2, and ß-ARs. These subfamilies can be further divided into subtypes of α1A, α1B, α1D, α2A, α2B, α2D, ß1, ß2, and ß3. All of these receptors are G-protein-coupled receptors (GPCRs) that require catecholamines such as adrenaline and noradrenaline for activation [4,5]. Different types of G proteins are responsible for signaling through different AR subfamilies. ß-adrenergic receptors are present predominantly in the brain, lung, liver, kidney, breast, ovary, and prostate. Ligand (norepinephrine and epinephrine) binding to GPCRs activates the stimulatory subunit of the heterotrimeric G protein, which results in the accumulation of cyclic AMP (cAMP) through the activation of adenylyl cyclase (AC) [6]. This elevated cAMP level can affect many cellular processes by acting through two main downstream systems. The first pathway is through cAMP-dependent activation of protein kinase A (PKA), which subsequently phosphorylates the target proteins. PKA regulates many cellular processes such as cell metabolism, growth, differentiation, morphology, secretion, and gene expression. PKA also activates the ß-adrenergic receptor kinase (BARK), which desensitizes ß-receptor signaling via activation of the Src/Ras/mitogen activated kinase (MAPK) pathway [6]. The second pathway involves another cAMP effector called the AC-activated guanine nucleotide exchange protein (EPAC). This protein activates Rap1A, a Ras-like triphosphatase that stimulates the downstream effector pathway of BRAF, MAP, and extracellular signal-regulated kinase (ERK)1/2. These two pathways overlap or complement each other at certain points to form the downstream system of cAMP (Figure 1) [7].

Physiologically, the SNS and its ligands have major roles in the regulation of cardiac and vascular adaptations via ARs expressed on endothelial surfaces, a detailed presentation of which was not within the scope of this paper. Therefore, for further details regarding normal functions of ARs, we refer readers to a recent comprehensive overview of the topic in [8]. We only briefly mention here that these receptors regulate endothelial functions by releasing nitric oxide (NO), which elicits vasorelaxation through the activation of cGMP-dependent protein kinase. Endothelial dysfunction has been associated with several disorders such as hypertension and type 2 diabetes mellitus [8]. These pathways are also responsible for the relaxation of smooth muscle cells through increased calcium reuptake into the sarcoplasmic reticulum and for hyperpolarization of these cells’ membrane within bronchi or veins. As all ß-ARs are present in brown and white adipocytes, these pathways are important in thermogenesis and lipolysis as well [9].

Interactions between the immune system and the nervous system are crucial in maintaining immune homeostasis. Both the central nervous system (CNS) and the peripheral nervous system (PNS) are involved in modulating the immune response. The autonomic cell groups of the CNS and PNS, which use sympathetic and parasympathetic neurotransmitters, generally regulate the internal homeostasis of the organism. Most tissues of human body are to some extent innervated by both. The neuro-immune interactions are primarily mediated by adrenergic ligands and receptors [10,11]. Autonomic nerve fibers end in lymphoid organs and have important immunomodulatory roles in several disorders (including cancer) [12]. However, sympathetic and parasympathetic nerve fibers can also end in tumor tissues, thereby conveying direct regulatory effects on cancer cells [13,14]. 

There are studies suggesting a link between stress-activated pathways and cancer progression because catecholamines exert direct tumor-promoting effects in numerous different cancer types that affect the breast, ovary, colorectal tissues, esophagus, lung, and prostate as well as in melanoma and leukemia [15,16,17]. The results of our recent studies added glioblastoma (GBM) to the list based on an established link between catecholamines and cancer. Our epigenomic and quantitative immunohistochemical studies showed that the pathways involved in catecholamine signaling are more active in primary than in recurrent tumors or controls, which suggested that these molecules play a role in the early phase of GBM development [18,19]. Tumor cells express ß-ARs not only in GBM (ß1-ARs and ß2-ARs) but also in many other tumor types, which can influence intracellular signaling involved in cellular replication, inflammation, angiogenesis, apoptosis, cell motility, DNA damage repair, and immune responses. 

In addition to normal tissues and tumor cells, ARs are also expressed on the surface of various immune cells that include neutrophils, monocytes, natural killer (NK) cells, macrophages, and mature dendritic cells, in which the ß2-AR is the most highly expressed AR subtype [20]. Through these receptors, catecholamines are able to act on hematopoietic stem cells or progenitor cells in the bone marrow, thus increasing the production of inflammatory cells, which in turn could modulate the tumor microenvironment and promote tumor growth [21,22]. However, ARs are also expressed on immune cells present in the tumor microenvironment (TME) [23]. Activation of these ARs has numerous different effects. Dendritic cells (DCs), which are responsible for antigen presentation, express both α-AR and ß-AR. The α-ARs are predominantly receptors that are associated with the stimulation of the immune response, while interaction with ß-ARs is typically accompanied by inhibition of the activity of the immune system. The presence of NE induces immune receptor stimulation, which in turn results in enhanced IL-10 secretion and decreased IL-12, IL-6, and TNF-α secretion, thereby leading to immunosuppression and impaired Th1 priming [24]. NE binding to its AR on CD8+ T cells inhibits cytotoxicity, while acting on CD4+ T cells reduces IL-2 production and enhances the suppressive function of Treg cells. NE acting on activated B cells may hamper antibody production. Regarding NK cells, NE inhibits migration and suppresses NK cells’ toxicity. Macrophages may also be modulated by NE by causing decreased phagocytic activity, while its action on monocytes results in immunosuppression via downregulation of TNF-α. 

While not strictly part of the present survey, it is worth noting that dopamine receptors are also expressed on the surface of immune cells; e.g., T and B lymphocytes, dendritic cells, macrophages, NK cells, and Treg cells. Dopamine, another catecholamine transmitter, has anti-inflammatory effects that primarily manifest in the suppression of macrophage functions, while its immunostimulatory effects via activation of resting/naive T cells and stimulation of the secretion of TNF-α and IL-10 through D2/D3 and D1/D4 receptors are also known [25]. Dopamine-induced stimulation of D1 receptors can downregulate the immunosuppressive activity of regulatory T cells and the production of IL-10 and TFG-ß, thus dopamine is able to exert an activating effect on the immune response by suppressing immunosuppression [24]. For a more detailed discussion of immune cells and AR, we refer readers to a recent review by Chhatar and Lal [26]. While a relatively large amount of information is available about immune cells and their receptors, the exact molecular mechanisms of adrenergic signaling in the development and progression of malignancies remain unclear [3]. 

In summary, the adrenergic system (and through it, various forms of stress) may affect tumor biology either via direct interactions between the SNS and tumor/TME, or via the SNS–immune system–tumor. Further, some tumor and TME cells can produce sympathetic neurotransmitters to autoregulate their biology via ARs expressed on their own surfaces. In this review, we focus on summarizing the most important and most recent data on the role of ß-ARs expressed on tumor cells and the TME in the development and progression of cancers.

## 2. Review of the Literature

### 2.1. ß-Adrenoreceptors and Breast Cancer

Breast cancer (BRCA) is the most frequently diagnosed cancer that affects women of all ages worldwide and is expected to represent around 25% of all new cancer cases in females. Despite the encouraging progress achieved in early diagnosis and treatment, 10% of breast cancer patients have metastasis at the time of diagnosis [27]. While the overall survival rate is reasonably high today (the average 5-year survival rate for women in the United States with nonmetastatic invasive breast cancer is 90%, and the average 10-year survival rate for women with nonmetastatic invasive breast cancer is 84%) [27], dissemination of BRCA cells to distant organs results in a poor prognosis [28]. Stress exposure has clearly been associated with carcinogenesis in BRCA [29,30]. Stress-related neurotransmitters (noradrenaline and adrenaline) can participate in the process via activation of their cognate receptors in TME or prompt systemic changes [31,32] that include the suppression of the immune system [33]. Adrenergic signaling may promote neoplastic transformation of breast epithelial cells in the initiation phase; however, it may also favor the metastatic potential of breast cancer cells during cancer progression. Although the presence of mesenchymal cells is essential for the induction of tumorigenesis, adrenergic signaling not only affects directly mesenchymal and tumor cells but also the function and activity of other cell types in the TME, where macrophages, fibroblasts, and fat cells can take on a kind of protumor phenotype [34]. Based on a previous BRCA cell line study, amongst all AR types, ß2ARs seem to be the most relevant receptors involved in the development of BRCA metastasis. ß2AR activation results in elevated intracellular cAMP and Ca2+ levels and reduces phosphorylated ERK, which activates a positive cAMP-calcium feedforward loop that drives breast cancer cell invasion [35]. 

The presence of mesenchymal cells is essential during breast tumorigenesis. These cells transform from cells of another phenotype (mainly epithelial cells) as part of epithelial–mesenchymal transition (EMT), a key mechanism in the genesis of many tumors [36,37]. Stress-induced adrenaline release is able to cause activation of EMT, while the pharmacological blockade of ß2-ARs inhibits the adrenalin-induced stem-like trait of breast cancer and thereby tumor growth [38]. The exact mechanism of tumor initiation is not fully understood, but the downregulation of miR-337-3p likely plays a vital role in it because this microRNA inhibits the activation of the signal transducer and activator of transcription 3 (STAT3) transcriptional factor. The downregulation of miR-337-3p is able to downregulate E-cadherin expression and upregulate EMT markers such as vimentin, which are the consequences of the stress-induced adrenergic activation of STAT3 [39]. 

Cell proliferation is a critical element of cancer progression. Previous studies have shown that chronic stress increases cancer cell proliferation and breast tumor growth through adrenergic stimulation [31,38]. Increased rates of cell proliferation have mainly been linked to the activation of ß2-ARs, which in turn activates the p38 mitogen-activated protein kinase (P38/MAPK) pathway and the protooncogene human epidermal growth factor receptor 2 (HER2) [40,41]. ß-AR activation also can contribute to breast cancer growth via activation of the lactate dehydrogenase/ubiquitin specific peptidase 28/MYC/SLUG signaling axis [38].

Breast cancer metastasis may be enhanced by adrenergic stimulation by activating the pathways involved in invasion and cell migration [38,39]. The mechanisms involved in tumor initiation and metastasis formation may be identical, while the adrenergic-induced EMT may also enhance the transformation of primary tumor cells into circulating tumor cells. However, since breast cancer cells are most likely capable of synthesizing catecholamines themselves, the formation of metastasis is presumably largely independent of adrenergic signaling [42]. Metastatic breast cancer cells preferentially target certain organs such as the lungs, liver, bones, and brain. Among these, involvements of bones and lung account for 40–70% of metastases [43]. Data suggest that the adrenergic system also targets these two organs by conditioning the local microenvironment for colonization by breast cancer cells. The activity of ß2-ARs results in the upregulation of chemokine ligand 2 (CCL2) in the lung stromal cells and the upregulation of its receptor (chemokine receptor type 2 (CCR2)) on the surrounding monocytes and macrophages, which together promote infiltration and colonization of breast cancer cells to the premetastatic space [44]. Stromal cells are multipotent mesenchymal cells (e.g., cancer-associated fibroblasts) with an immunomodulatory capacity exerted by direct cell-to-cell contact and by paracrine secretion of soluble factors. Thus, stromal cells may influence the activity of NK cells, repress the activation of dendritic cells, modulate the proliferation of B cells, and induce expansion of regulatory T cells. Apart from the indirect modulatory effects through immune cell components, stromal cells can directly influence tumor growth via cell-to-cell contacts and soluble molecules [45,46]. Similarly, the activity of ß2-ARs contributes to the metastatic processes in the bone marrow as well. An increase in the expression of the receptor activator of nuclear factor kappa-B ligand induces the promigratory activity of breast cancer cells, which ultimately leads to bone colonization [47].

### 2.2. ß-Adrenoreceptors and Lung Cancer

Lung cancer, which is the most common cancer and the leading cause of death for both sexes, has an overall 5-year survival rate of less than 20% [48]. Similar to those reported for several other types of cancer, epidemiological studies in lung cancer have shown that ß-AR signaling has an effect on the development and progression of the disease. The use of ß-AR blockers on ß2-ARs appears to be associated with better clinical outcomes in non-small-cell lung cancer (NSCLC), which is similar to their protective effects in other cancers. ß-ARs can play significant roles in the biology of lung cancer because they are expressed to varying degrees in normal bronchial epithelial cells and in NSCLC cell lines [49]. 

As described in breast cancer, the tumorigenesis-inducing effect of chronic stress has also been demonstrated in lung cancer. In a mouse model, chronic stress promoted Kras^G12D^ mutation-induced tumorigenesis through elevated catecholamine (mainly NE) synthesis. An increased NE level causes ß2-AR activation, which in turn generates voltage-dependent Ca2+ channel-mediated Ca2+ influx. The elevated level of intracellular Ca2+ induces activation of the insulin-like growth factor 1 receptor (IGF-1R) via release of insulin-like growth factor 2 (IGF2) from airway epithelial cells, which leads to the transformation of the lung epithelial cells and to tumor initiation [50]. Kaira and colleagues also confirmed the role of ß2-ARs by investigating pulmonary pleiomorphic carcinoma (PPC), a rare and aggressive type of lung cancer. The authors demonstrated ß2-AR expression in 63% of the tumors, and the expression levels of ß2-AR significantly and positively correlated with the tumor′s cell proliferation rate in surgically resected PPC specimens [51]. 

### 2.3. ß-Adrenoreceptors and Melanoma

The number of skin cancer cases has been continuously increasing in recent decades. Melanoma is one of the most aggressive and the least chemotherapy-responsive human cancer type. Although many forms of targeted and immunotherapies have recently changed the prognosis of several tumors (including melanoma) that typically have a poor prognosis, early diagnosis and treatment are still crucial for long progression-free and overall survival [52]. In melanoma, similarly to other tumors, the activation of the SNS results in a local release of catecholamines (NE and E) along with the activation of the ß-AR signaling cascades that ultimately lead to the formation and progression of the tumor as well as the adjustment of the TME. In skin cancers, especially in melanoma, all ß1, ß2, and ß3-ARs are expressed, of which ß1 appears to be the most significant. It is worth noting that among all tumor types studied, ß-ARs showed the highest expression in melanoma, which provided convincing evidence for the relationship between the adrenergic system and this skin cancer type [53,54].

In melanoma, as in other tumors, the previously mentioned signaling pathways (cAMP/PKA, PI3K/Akt, Ras/ERK1/2, and P38/MAPK) are responsible for tumor development and progression. However, in addition to ß1- and ß2-ARs, the role of ß3-ARs in the regulation of cell proliferation has also been demonstrated. It appears that ß3-ARs uniquely play an essential role in influencing the proliferation of melanoma cells through nitric oxide signaling [3,55]. We need to note here a well-known metabolic feature of cancer cells: the so-called Warburg effect. Normal, differentiated cells rely on mitochondrial oxidative phosphorylation to generate energy, while cancer cells preferentially rely on glycolysis even in the presence of oxygen (this process is called aerobic glycolysis or the Warburg effect). The latter is a highly inefficient way to generate ATP, and the advantage it confers to cancer cells has been unclear [56]. It was recently demonstrated that ß3-ARs induce a metabolic shift with accelerated glycolysis and reduced mitochondrial activity through the induction of uncoupling protein 2 (UPC2) in both melanoma and embryonic stem cells. This ß3-AR/UPC2 signaling axis results in a significant decrease in ATP synthesis and an increase in the mitochondrial reactive oxygen species (mtROS) [52]. ß3-ARs are present in the mitochondrial membrane of melanoma cells, in which they affect mitochondrial dormancy. In addition, ß3-ARs are also expressed in inflammatory and vascular cells in vivo in the microenvironment of melanoma [57]. The simultaneous presence of these accessory cells that express ß3-ARs and melanoma cells in the same microenvironment results in stronger cellular responses of melanoma cells to macrophages and stromal fibroblasts via ß3-ARs (which is preventable by ß3-AR selective silencing), thus helping to endow melanoma cells with stem-cell-like properties. In addition, ß3-AR activation in these cells will induce the secretion of inflammatory cytokines, which will promote tumor growth through angioneogenesis. Because ß3-AR plays a major role in inducing the motility of melanoma-related cells, this receptor type is likely involved in metastasis [58]. Cell line experiments demonstrated that A375 melanoma cells are able to induce the migration of macrophages, fibroblasts, monocytes, and endothelial cells; however, this can be blocked with a ß3-AR selective inhibitor SR59230A by increasing mtROS but not with ß2-AR selective silencing [59]. 

### 2.4. ß-Adrenoreceptors and Gliomas

Malignant glioma, which is the most common primary brain tumor, accounts for approximately 80% of tumors in the CNS. Glioblastoma (GMB), a grade IV glioma, is characterized by exceptionally rapid proliferation, aggressive growth, strong intra- and inter-tumor heterogeneity, high recurrence rates, and a poor prognosis. Therefore, it is important to understand the regulatory mechanisms behind these characteristics. Currently, the basis of GBM treatment is still surgical removal of the tumor followed by adjuvant chemotherapy and radiation therapy (the STUPP protocol) [60]. While several personalized molecular and immune treatment strategies have been tried in high-grade gliomas, their benefit thus far appears limited [61,62,63,64].

The involvement of catecholamines (specifically NE) via the activation of ß-ARs and the cAMP/PKA signaling pathway has been proven in other cancers (as discussed above and in other papers) [65,66,67]. Meanwhile, chronic-stress-related glioma studies are rare. Nevertheless, some investigations have shown changes in the expression of certain genes and signaling pathways that may contribute to the formation and development of gliomas. The predominant signaling pathways involved in glioma cell regulation include the RAS/RAF/ERK, p53/MDM2/p21, and PI3K/Akt/mTOR pathways initiated by various growth factor receptors (which generally belong to the receptor tyrosine kinase molecular family) [68,69]. Notably, catecholamines can also alter these downstream signaling pathways through ß-ARs. Our group recently sequenced bisulfite-converted DNA libraries and analyzed genome-wide patterns of DNA methylation in GBM. We detected several differentially methylated pathways from surgically removed, formalin-fixed, paraffin-embedded (FFPE) human GBM sample pairs obtained at diagnosis and recurrence. Among them, the pathways of catecholamine secretion and transport appeared hypomethylated in the primary compared to the recurrent tumors. This observation prompted us to simultaneously determine the expression levels of four catecholamine markers and their promoter + gene methylation levels in individual GBM samples. Our results revealed that protein expression levels of the four selected markers, namely α1D-adrenergic receptor (ADRA1D), adrenergic ß-receptor kinase 1 (ADRBK1), dopamine receptor D2 (DRD2) and vesicular monoamine transporter 2 (VMAT2), were significantly or tendentiously higher in primary compared to recurrent GBM. To determine how the expression levels of the markers are influenced by the CpG methylation levels in their promoters and genes, we quantified protein expression via immunohistochemistry (IHC) and CpG methylation in the promoter + gene regions using a bioinformatics script for the four markers in 21 pairs of GBM samples and in control brain samples. These analyses showed either significantly (ADRBK1 and DRD2) or tendentiously (ADRA1D and VMAT2) higher methylation levels along with lower protein expression levels of these markers in the control brain samples compared to either the primary or recurrent tumors, thereby reflecting the expected inverse correlation between gene methylation and protein expression. However, we did not find differences in the methylation levels in our primary and recurrent GBM samples or in a similarly studied database cohort of primary and recurrent GBM samples. Nevertheless, the protein expression levels of the four markers appeared higher in our primary than in recurrent GBM samples, which underscored that CpG methylation is an important (but not exclusive) mechanism responsible for gene expression regulation. The higher marker protein expression in the primary tumor samples suggested that the catecholamine pathway may be more active in earlier phases of tumor development. Altogether, our observations suggested that catecholamine neurotransmitters, their receptors, and their signaling molecules may play important roles in gliomagenesis and likely drive the onset, development and (to some degree) growth of GBM [18,19]. 

Based on the results of cell line experiments, an earlier study demonstrated that ß-adrenergic signaling plays a role in the proliferation of tumor cells. Jing He et al. investigated the glioma cell lines U251 and U87-MG and demonstrated the expression of ß1-ARs and ß2-ARs [70]. The activation of these ß-ARs by isoproterenol (ISO) (a nonselective β-AR agonist) resulted in a significantly enhanced cell proliferation rate in U251 cells via activation of the ERK1/2 pathway and mRNA expression of matrix metalloproteinases (MMP)-2 and MMP-9, which potentially facilitated cell migration and metastasis formation. Another signaling pathway known as the AC pathway may also be important in gliomas because its activation results in an intracellular accumulation of cAMP. The elevated cAMP level may play a role in either promoting or suppressing cell proliferation (depending on the malignant cell types). There are two theories regarding cAMP signaling. The first theory states that an increase in intracellular cAMP levels suppresses cell proliferation in most mesenchymal and epithelial cell lines (including GBM) [71]. In contrast, the other theory claims that cAMP promotes cell survival, which can be observed in many cell types such as myeloid cells, hepatocytes, dopaminergic neurons, gastric cells, and intestinal cells [72]. In an elegant study, Safitri et al. demonstrated that elevated intracellular cAMP levels induced by the AC activator forskolin suppressed growth of the C6 glioma cells. The antiproliferative effects of forskolin are mediated by cAMP-activated PKA and by cell cycle arrest in the G2/M phase. The authors also tested a range of small-molecule phosphodiesterase (PDE) inhibitors with differing selectivity profiles. Phosphodiesterases are the enzymes that hydrolyze cAMP synthesized by AC within cells. In comparison with forskolin, the inhibition of PDEs by trequinsin not only inhibited cell growth via the cAMP/PKA cascade but also triggered cell death. Finally, concomitant targeting of both AC and PDEs synergistically elevated intracellular cAMP levels, thereby potentiating their antiproliferative actions [73]. 

The link between chronic stress and cancer progression with the involvements of catecholamines that exert direct tumor-promoting effects has been demonstrated in numerous cancers. Whether or not chronic stress and the signaling pathways activated by catecholamines affect glioma behavior remains to be further explored because only scarce data are available. Zhang et al. performed animal studies and cytological analyses to investigate the effects and molecular mechanisms of chronic stress in glioma cells [74]. The results showed that circulating glucocorticoid (GC) and NE levels in serum were significantly increased in vivo under stress and promoted glioma growth in U87MG cells, which were injected subcutaneously into mice. IHC staining revealed that Ki67 expression was higher in the stress group, thereby indicating that chronic stress could enhance tumor proliferation and burden in the mouse model. The stress hormones GC and NE were also proven to accelerate the proliferation of glioma cells in vitro using U87MG and LN229 glioma cell lines. The proliferation rate of GC- and NE-treated cells was not only increased, but these cells also formed more colonies. Furthermore, the authors found that the expression levels of p-PI3K and p-Akt in U87MG and LN229 cells were increased under the influence of GC and NE, thereby indicating activation of the PI3K/Akt pathway and thus showing its potential regulatory role in the chronic-stress-induced glioma proliferation [74]. Another recent study also addressed the effects of catecholamines—specifically EN—in glioma cell lines. The effects of EN in different concentrations were investigated for cell adhesion, cytotoxicity, and viability in U87 cells. The results showed that at a physiological concentration, EN accelerated proliferation but prevented metastasis. However, in a pharmacological concentration, oxidative stress resulted in hydrogen peroxide and ROS production that inhibited cell proliferation and promoted metastatic capacity of the tumor. Apparently, EN acted as a double-edged sword [75]. Using subcellularly targeted ERK activity biosensors, Kwon et al. investigated ß2-AR signaling known to involve activated ERK, a regulator of cell proliferation and survival. The authors demonstrated that the ß2-ARs-induced ERK activity was not localized to the plasma membrane but to the endosomes. This type of ERK activity depended on active endosome-localized G-alpha subunits and required ligand-stimulated ß2-AR endocytosis. Furthermore, selective ERK inhibition of these endosomal signaling axis with SCH772984 blunted the nuclear ERK activity and thereby cell proliferation [76]. 

In addition to the high cellular proliferation rate, the genetic instability, chromothripsis, high angiogenesis, and diffuse tissue invasiveness are also important characteristics of GBM. Using fluorescence and confocal microscopy, Pavlova et al. examined glioma cell migration from the main tumor mass and cerebrovascular changes associated with glioma growth. The study revealed that glioma cells used the cerebral vessels and infiltrating macrophages in the tumor mass to increase the permeability of the blood–brain barrier (BBB) via a loss in astrocyte mediated gliovascular coupling and tight junctions, thereby resulting in a focal breach in the BBB. When the ß2-ARs were stimulated by isoproterenol, an early migration of glioma cells and a decreased survival of animals were observed. In contrast, pharmacological blockade of ß2-ARs by ICI-118551 reduced glioma cell migration and increased the survival rate of the animals, which suggested that manipulation of the ß2-AR signaling pathway may improve the efficacy of glioma therapies [77].

Table 1 provides a brief overview of available knowledge concerning β-AR subtypes, signaling elements, and pathways as well as their biological effects in the TME of the four surveyed tumors. 

### 2.5. ß-Adrenergic Signaling Regulating Immune Components in the TME of Different Tumors

The ß-adrenergic system may modulate tumor biology as a significant regulator of the immune system in the TME. Catecholamines are able to bind to ß2-ARs on white blood cells and inhibit lymphocytic responses, NK cytotoxicity, and dendritic cell function [15]. Nevertheless, relatively little is known about the specific roles of the ß-adrenergic system on immune cells in the TME.

In breast cancer, a high density of M2 macrophages usually indicates a poor prognosis. In a mouse model of breast cancer, epinephrine significantly induced macrophage transformation to the M2 phenotype. We have relatively little knowledge regarding how adrenergic signaling shapes the TME in human breast cancer. Earlier studies demonstrated that norepinephrine-activated ARs (α2-AR and ß1-AR) can regulate TNFα and NO release in macrophages through various signaling pathways [78,79,80]. A more recent study revealed that high ß2-AR expression level was associated with a low level of tumor-infiltrating lymphocytes in estrogen receptor (ER)-negative breast cancer, which suggested that high ß2-AR expression is a poor prognostic factor [81]. 

In in vitro lung cancer studies, catecholamine treatment drove macrophages toward a tumor-supportive M2 polarization via adrenergic signaling while also enhancing the expression of vascular endothelial growth factor (VEGF), thereby promoting tumor angiogenesis in the HCC827 human NSCLC cell line and the H446 small-cell lung cancer cell line. Of note, these catecholamine-stimulated effects could be reversed by propranolol. In contrast, decreasing the catecholamine levels shifted the immunosuppressive microenvironment by decreasing MDSC recruitment and facilitating the activation of DCs with the potential of inducing a positive antitumor immune response [82].

Melanoma is a tumor that is highly sensitive to immune therapeutic agents. The TME of melanoma is enriched in tumor-associated M2 macrophages, Treg cells, and myeloid-derived suppressor cells (MDSC), which may negatively regulate cytotoxic T cells [83,84]. Nevertheless, little is known about the role of ß-ARs in regulating the immune environment in melanoma. The sparse evidence that exists suggests that ß-AR stimulation recruits and polarizes macrophages [85] that promote tumor progression. Furthermore, ß2-AR blockade improves the antitumor efficacy of immune checkpoint inhibitors or other forms of immune therapy (IL-2, αCTLA-4, and/or αPD-1) [86]. 

In gliomas, astrocytic ß2-ARs have emerged as potential regulators of CNS inflammation. Laureys et al. generated proinflammatory conditions via the administration of TNF-α and confirmed its relevance as a modulator of brain inflammatory responses and cell homeostasis via parallel transcriptional patterns in vivo and in vitro in the human 1321 N1 astrocytoma cell line. Upon TNF-α treatment, the upregulation of the expression of several immune regulatory genes that included IL-6, CXCL2, CXCL3, VCAM1, and ICAM1 was noted, which suggested their roles in inflammatory brain cell homeostasis [87]. Although many types of immune cells express ß-ARs, there is still little knowledge about their exact role in shaping the TME and immune evasion. 

### 2.6. ß-Adrenoreceptors and Potential Treatment Options

The global cancer burden is continuously increasing. Estimates of the worldwide distribution of cancer cases are approaching 20 million, a figure that can be further broken down among the most frequent tumor types (breast, lung, colorectal, prostate, stomach, liver, cervix, uterine, and other cancers) [88].

The treatment protocols are different, but in general, surgical removal of the tumor, chemotherapy, and radiation therapy are the main options; these have only recently been complemented by personalized molecular and immune therapies. The spectrum of cancer control interventions ranges from primary prevention, screening, and early diagnosis to palliative care [89]. 

Due to their β-AR antagonist effects, beta-blockers (BBs) are commonly used in various diseases such as hypertension or coronary artery disease [90]. However, the above survey supported that BBs may be (and even have been) considered for cancer treatment due to their antagonistic effects on adrenergic receptors involved in triggering tumorigenesis, angiogenesis, and metastasis. Based on their affinity toward ß-ARs, BBs are subclassified as ß1-selective (cardioselective; e.g., atenolol, celiprolol, bisoprolol, etc.) or nonselective if acting on both ß1- and ß2-ARs (e.g., propranolol, sotalol, carvedilol, etc.). The use of BBs has emerged as a possible option for prolonging relapse-free survival and overall survival (OS) in the treatment of many tumor types [91]. As mentioned above, the induction of catecholamines is linked to the activation of genes associated with metastasis, inflammation, activation of cell proliferation pathways, and upregulation of proangiogenic factors. Considering that these mechanisms are executed partly via ß2-ARs, nonselective BBs such as propranolol could be (on a theoretical basis) a promising anticancer agent. Disappointingly, however, a meta-analysis concluded that the use of propranolol did not result in any significant differences in the cancer-specific death rate, overall death rate, or relapse-free survival rate compared to nonuse [92]. 

An earlier BRCA population study suggested that the use of BBs during chemotherapy helped to improve relapse-free survival but not OS [93]. Patients who were already taking BBs for some reason before their BRCA diagnosis had a significantly lower rate of metastases [94]. In another explorative study, patients with stage III HER2-negative BRCA were treated with propranolol for 18 days. Subsequently, tumor tissues were collected and Ki-67 expression levels were evaluated. With propranolol treatment, both Ki-67 and Bcl-2 (a prosurvival, antiapoptotic marker) expression decreased, but p53 protein expression increased. These findings were validated in an MDA-MB-231 breast cancer cell line exposed to propranolol and doxorubicin; this reduced the rate of cells arrested in the G2/M phase while cells died or were dying. These results supported the assumption that BBs do have antagonistic effects on BRCA cell proliferation and may lead to apoptosis, thereby inhibiting tumor growth and development [95]. In another study, expressions of ß1-, ß2-, and ß3-ARs were quantified ex vivo in normal breast and BRCA tissues. ß1 and ß3 receptors were more highly expressed in cancer tissue; however, ß2 expression showed no difference. Cells collected from BB-user patients with stage I and II BRCA showed a significant decrease in Ki-67 expression compared to those from nonusers. To corroborate these results, the authors treated patients with HER-2 negative BRCA using propranolol. The Ki-67 index was evaluated before and after 25 days of treatment. The post-treatment Ki-67 expression was 23% lower than the pretreatment levels, which suggested that propranolol may significantly decrease tumor proliferation [96].

Lung cancer studies have shown partially contradictory results. Lin et al. demonstrated that the long-term use of carvediol was associated with a reduced risk of lung cancer, which suggested a potential role for ß-AR blockade in cancer prevention [97]. There have been numerous retrospective studies that evaluated the impact of BB use on clinical outcome in patients with NSCLC. Some of these population-based cohort studies showed no association between BB use and reduced mortality among lung cancer patients [98,99,100]. However, others indicated that BB use was indeed associated with an improved outcome because BB use during chemotherapy treatment improved OS in patients with metastatic NSCLC [101]. Another recent retrospective study evaluated the effects of propranolol on the metastatic rate of NSCLC and survival rate of effected patients as well as the clonogenic viability of two human lung adenocarcinoma cell lines (PC9 and A549) in cultures. Statistical testing demonstrated that BB use was associated with decreased distant metastases and potentially improved OS and distant metastasis-free survival; however, it did not affect the primary tumor pathology in patients treated for stage IIIA NSCLC [102]. These results suggested that BBs may have a role in inhibiting metastasis rather than in controlling cell growth in the primary tumor.

To date, no formal BB clinical trial results have been collected in patients with melanoma. Nevertheless, some evidence of the effectiveness of BBs in melanoma has emerged from the early observations of De Giorgi et al. [103]. The examined cohort included 30 BB users and 91 untreated patients who were followed for a median of 2.5 years. Tumor progression was observed in 34.1% of the untreated subgroup but only in 3.3% of the treated subgroup [103]. This difference remained noticeable even after a longer follow-up of 8 years, when 30% of the patients in the treated group and 45% in the untreated group showed disease progression [104]. In an even more recent prospective study, patients were asked to take propranolol at diagnosis. Of the 53 patients, 19 entered into the propranolol cohort and 34 into the non-propranolol cohort. After a 3-year follow-up, the authors noted that the use of propranolol was inversely associated with the recurrence of melanoma and reduced the risk by about 80% [105].

Regarding gliomas, there is limited information about the effects of BBs. Pavlova et al. injected rats with rat C6 glioma cells into the caudate putamen area and treated half of them with ICI-118551 (a specific ß2-AR antagonist) starting from the day of the implantation. The control rats received either saline or isoproterenol. The rat group that received the ICI-118551 BB survived significantly longer (a median of 45 days) compared to the control group (a median of 20 days). Confocal imaging revealed that the ß2-AR blockade decreased glioma cell migration by 20% and reduced the disruption of the BBB significantly [77]. Johansen et al. examined the effect of BBs on recurrent GBM in a retrospective cohort of 218 patients. Two patient groups were compared, one of which received BBs while the other received no AR-modulating drug. Both groups were treated with bevacizumab. Unfortunately, no association was found between BB usage and OS or progression-free survival (PFS) [106].

When surveying the treatment effects of ß-blockers, the concept of biased agonism needs to be briefly discussed. Biased agonism refers to the ability of a ligand to activate various subsets of signaling cascades of a receptor, which may lead to different biological effects. For example, carvediol (a nonselective BB) is a biased agonist of ß-ARs that is able to induce either ß-arrestin mediated signaling or G-protein subunit mediated signaling events [107]. In the case of balanced agonists, blocking G-protein signaling through cardiac ß1-ARs has a reductive effect on the heart rate and leads to an improved ejection fraction, while a ß-arrestin biased agonist can further improve the efficacy of this effect by desensitizing ß1-ARs at the cell membrane via internalization and transactivation of epidermal growth factor receptors [108]. In contrast, a G-protein biased agonist exerts its effects through ß2-ARs, which could provide enhanced bronchodilatation in smooth muscles of the airways in asthma, but the long-term use of these drugs may lead to serious adverse effects [109]. These multifaceted effects complicate the treatment of tumors with BBs because these drugs are not exclusive antagonists of G-protein pathways but can independently regulate several pathways by acting as partial agonists, inverse agonists, or pure antagonists. As a result, their effects are often confounded by this complexity [3]. A recent study highlighted the importance of conformational change kinetics on ß-arrestin using an in vitro single-molecule fluorescence system for examining the transmembrane (TM) unit VII of the ß2-AR. The authors compared an agonist (formoterol) and a comparatively ß-arrestin biased agonist (isotharine). The latter prolonged the dwell time of the active conformation of TM unit VII compared to formoterol, which suggested that ligand-dependent changes were contributing factors to the biased signaling and should be considered in future drug-discovery trials [110]. In another study, the role of G-protein receptor kinases (GRKs) was demonstrated in biased agonism at the ß2-ARs. A single point mutation of tyrosine 219 in TM unit V was found to convert the ß2-AR into a G-protein biased receptor. The mutated receptor showed a negligible level of agonist-promoted phosphorylation of GRK5/6 sites, a reduced level of phosphorylation of GRK2 sites, and comparable phosphorylation of PKA sites compared to the wild type of the receptor. These data demonstrated that a reduced amount or complete absence of GRK interaction with β2-AR is sufficient to result in distorted signaling in the G protein. The data emphasized the importance of these kinases in regulating biased signaling [111,112]. Despite the great potentials of these biased agonists, their varying effects have not been addressed in clinical studies or in routine care. These limitations are likely related to the difficulties in ligand identification and the need for head-to-head examination of selectively activated pathways along with possible physiological consequences that are induced by the activation of certain signaling pathways over others [112,113].

Nevertheless, we can establish overall that repurposing already approved drugs such as BBs for the prevention and suppression of cancer is a very attractive approach from individual, public health, and economic points of view. The available basic science and clinical research data provide evidence to support the role of the ß-AR pathways in the pathogenesis of cancer and the clinical benefits of manipulating these pathways. However, several questions remain to be answered regarding (1) what molecular characteristics define the variability of the ß2-AR pathway involvements in various histological types of cancer; (2) what effects are mediated by CNS and PNS sympathetic/parasympathetic neurons that affect a tumor and its microenvironment or the immune system or are mediated by ß2-ARs and ligands expressed in a tumor and the TME; (3) what subclasses of BB drugs are the most effective in various histological tumor subtypes; and (4) to what degree the manipulation by ARs may contribute to tumor suppression, when they should be administered during the disease course, and how they may complement standard oncology treatment protocols.

It is also worth mentioning that modulation of the ARs and pathways is not only feasible with traditional receptor agonists and antagonists. Chemogenetics arose recently as a potential future treatment strategy that combines pharmacological and genetic approaches. This approach theoretically may manipulate tumor growth by targeting sympathetic neurons (located in the brain or in the periphery) with projections to the site of the cancer or by directly targeting receptors in the tumor and TME. In chemogenetics, genetically modified cells are engineered to express designer receptors exclusively activated by designer drugs (DREADDs). For example, neurons with mutant G-protein-coupled muscarinic acetylcholine receptors respond to the synthetic drug clozapine-N-oxide (CNO) instead of acetylcholine [114,115,116]. A G_q_-coupled DREADD (hM3Dq) can be used to enhance neuronal activity, whereas another G_i/o_-coupled DREADD (hM4Di) may inhibit neuronal activity [116].

Following a similar paradigm, Zhang et al. reported four DREADD-related structures, thereby widening the list of potentially usable targets in future chemogenetic therapies while also revealing key details of the molecular basis for activating these receptors [117]. Chemogenetics as a therapeutic approach has already been implemented in the manipulation of important receptors in certain cancer models, but the number of publications is still very low and the data are preliminary. Ojima et al. successfully developed a method for direct activation of the metabotropic glutamate receptor 1 (mGlu1) via coordination-based chemogenetics (dA-CBC) using a palladium metal complex to activate the mutant mGlu1 in the brain. The authors also worked out a strategy for cell-type-specific mGlu1 activation using adeno-associated viruses that carried a mGlu1 mutant [118]. These results showed that certain receptors can be precisely targeted to desired cell types. 

As mentioned above, sensory neurons may also be able to influence cancer progression. In an elegant study, DREADDs were used to manipulate the activity of sensory neurons in a Nav1.8-Cre mouse model of melanoma. Silencing of the activity of sensory neurons triggered increases in melanoma growth and intra-tumoral angiogenesis, while chemogenetic stimulation of these neurons inhibited melanoma progression via regulating immune surveillance, angiogenesis, and tumor growth [119]. Activation of dopaminergic neurons in the ventral tegmental area by DREADDs enhanced the antibacterial activity of monocytes and macrophages and thus reduced the bacterial load in a mouse model of delayed-type hypersensitivity [120]. Following these results, the authors activated the dopaminergic reward cells using DREADDs in the C57BL tumor-bearing mouse model, which significantly reduced the growth of the implanted Lewis lung carcinoma and caused a 50% weight reduction in B16 melanoma [121]. This experiment provided a very useful paradigm for targeting tumors via selected receptors using chemogenetic intervention. In this line, CNO, an already accepted and administered compound with no significant side effects in humans, could be used to control cancer. For the application of chemogenetics in human cancer, the selection of the desired neuronal groups (e.g., those involved in stress response) or cancer cells (that are to be directly targeted), genetic manipulation of the exact receptor and ligand combinations, and definition of the proper timing will all be essential [114,115]. While in a very early stage of development, this receptor-specific approach has a great potential for future implementation in precision oncology.

## 3. Summary and Conclusions

**Figure 2:**
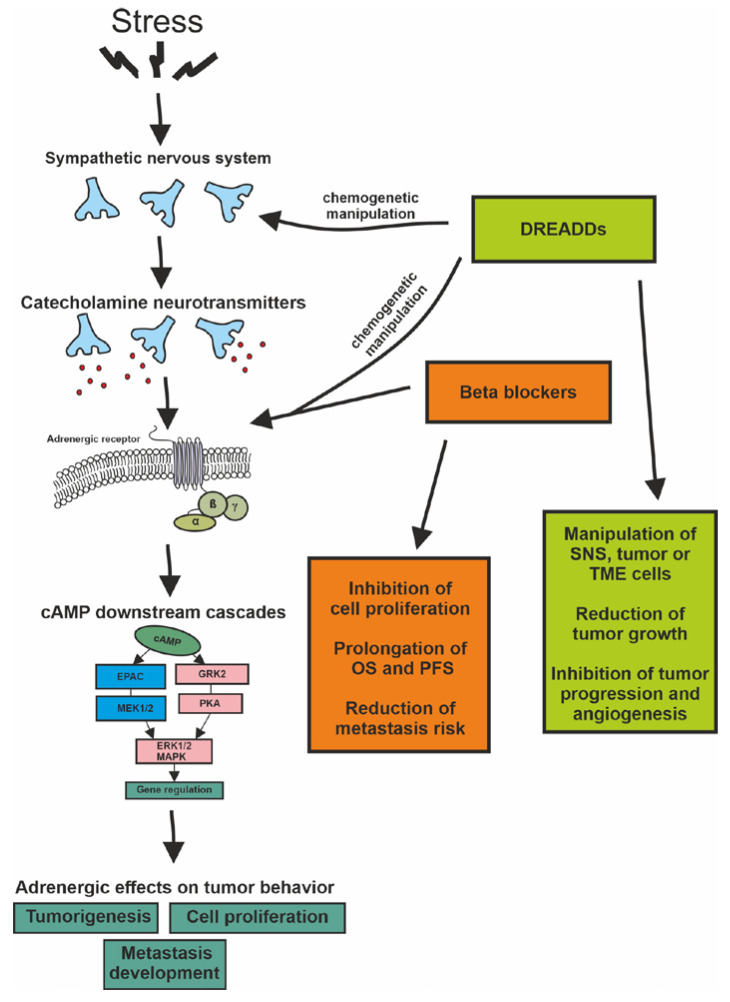
The effects of stress hormones via ß-adrenergic signalization pathways and the potential treatment targets of beta-blockers and DREADDs. Figure 2 shows the signaling pathways involved in stress response via ß-ARs the and potential modulation of the pathway by BBs or genetic engineering known as chemogenetics.

Cancer development is influenced by multiple factors that include genetic susceptibility, age, lifestyle, and numerous environmental toxins. All of these elements converge as the somatic accumulation of DNA point mutations, chromosomal rearrangements, small- and large-scale deletions/insertions, and gene copy number variations, as well as epigenomic changes that underlie gene expression regulation or metabolic shifts, all of which are mechanisms that profoundly alter cell biology. In addition to these well-known contributors to cellular transformation, chronic stress may also play an important role in the formation and development of tumors via stress-activated signaling pathways (Figure 2). 

Our body fights stress by activating catecholamine neurotransmitters in the SNS. These signals exert their effects through the well-characterized α- and ß-ARs that are expressed in most tissues. In breast and lung cancer, melanoma, and gliomas, a great body of observations supports that ß-ARs are involved the downstream systems of cAMP and can play a prominent role in tumorigenesis by maintaining cell proliferation and contributing to the development of metastases. Nevertheless, questions remain regarding how profound the involvement of ß-AR activation or blockade is in cancer biology due to the ambiguous observations of the effects of ß-AR blockers in human malignancies. The activation of catecholamine receptors and their downstream pathways likely is not alone sufficient to trigger but rather only modulates tumor initiation and growth through multiple mechanisms that involve the sympathetic nervous system or the tumor initiating cells, the TME, and immune regulatory cells directly. Experimental results from both in vivo and in vitro studies have demonstrated the effectiveness of BBs in the surveyed tumor types, but this mostly meant only a certain level of inhibition in the proliferation of tumor cells. In vivo, BBs could confer the benefits of prolonging OS or PFS in patients and reducing the risk of metastasis, but they did not significantly affect the pathological properties of the tumor itself. Nevertheless, the data overall support that tumorigenesis may be influenced by targeting the ß-ARs, thereby offering a complementary intervention. While the most effective anticancer BB drugs and their routes of administration and timing remain to be identified in each histological tumor type, there are also some newer groups of drugs and approaches that hold great promise in the receptor-specific manipulation of cell biology. These new directions include chemogenetics, which may very precisely and effectively target desired receptors and their downstream signaling pathways, thereby shaping the activity of a selected cell group (e.g., neurons or tumors) via an engineered DREADD. While BBs may not provide a sufficient advantage as a monotherapy and only act as adjuvants to the traditional anticancer approaches, the rapidly developing and increasingly tested chemogenetic paradigm may fulfill the hopes attached to them; namely, a very precise, selective manipulation of desired cells and their key receptor(s), which likely will represent a new direction in precision oncology.

## Figures and Tables

**Table 1:** Elements of the adrenergic signaling pathways in different tumor types. (Abbreviations: HER2, human epidermal growth factor 2; VEGF, vascular endothelial growth factor; STAT3, signal transducer and activator of transcription 3; FGF-2, fibroblast growth factor 2; Ang-2, angioprotein-2; MDM2, mouse double minute 2 homolog/E3 ubiquitin-protein ligase; LDHA, lactate dehydrogenase A; USP28, ubiquitin-specific peptidase 28; SNAI2/SLUG, snail family transcriptional repressor 2; iNOS, inducible nitric oxide synthase.

Tumor Type	Receptor Subtype	Adaptor Protein	Second Messenger	Signaling Elements	Effect on Cell Physiology
Breast	α2, ß1, ß2 more significant	Gα_s_, G_i_/G_o_	cAMP, Ca^2+^	MAPK, HER2, ERK1/2, LDHA/USP28/MYC/SLUG, PI3K/AKT/mTOR, VEGF, STAT3	Increased proliferation, metastatic rate, angiogenesis, and inflammation
Lung	ß1, ß2 more significant	Gα_s_	cAMP, Ca^2+^	PKA, EPAC, ERK1/2, MAPK, IGF-1R	Increased proliferation, immune evasion, angiogenesis, migration, and invasion
Melanoma	ß1 most significant, ß2, ß3	Gα_s_	cAMP, PKA, Ca^2+^	VEGF, P38/MAPK, PI3K/AKT, STAT3,iNOS, FGF-2, IGF-1, Ang-2, Ras/ERK1/2	Increased angiogenesis, proliferation, activation of stromal and inflammatory cells of TME, invasion, reduced apoptosis, and stem-cell trait induction
Glioma	ß1, ß2	Gα_s_	cAMP, PKA	ERK1/2, MAPK, PI3K/AKT/mTOR, p53/MDM2/p21	Increased proliferation rate, cell migration, angiogenesis, invasiveness, and metastasis formation

## Data Availability

Only published research data were surveyed and included in this manuscript.
